# The building blocks of embryo models: embryonic and extraembryonic stem cells

**DOI:** 10.1038/s41421-025-00780-6

**Published:** 2025-04-22

**Authors:** Hongan Ren, Xiaojie Jia, Leqian Yu

**Affiliations:** 1 State Key Laboratory of Organ Regeneration and Reconstruction, Institute of Zoology, Chinese Academy of Sciences, Beijing, China; 2 Beijing Institute for Stem Cell and Regenerative Medicine, Beijing, China; 3 University of Chinese Academy of Sciences, Beijing, China; 4 State Key Laboratory of Animal Biotech Breeding, College of Biological Sciences, China Agricultural University, Beijing, China

**Keywords:** Embryonic stem cells, Pluripotency

## Abstract

The process of a single-celled zygote developing into a complex multicellular organism is precisely regulated at spatial and temporal levels in vivo. However, understanding the mechanisms underlying development, particularly in humans, has been constrained by technical and ethical limitations associated with studying natural embryos. Harnessing the intrinsic ability of embryonic stem cells (ESCs) to self-organize when induced and assembled, researchers have established several embryo models as alternative approaches to studying early development in vitro. Recent studies have revealed the critical role of extraembryonic cells in early development; and many groups have created more sophisticated and precise ESC-derived embryo models by incorporating extraembryonic stem cell lines, such as trophoblast stem cells (TSCs), extraembryonic mesoderm cells (EXMCs), extraembryonic endoderm cells (XENs, in rodents), and hypoblast stem cells (in primates). Here, we summarize the characteristics of existing mouse and human embryonic and extraembryonic stem cells and review recent advancements in developing mouse and human embryo models.

## Introduction

Early embryonic development events, such as blastocyst formation, peri-implantation, implantation, gastrulation, and body axis establishment, are key areas of interest in current biological research. However, the specific events, key cell fate determinations, and underlying molecular mechanisms are still unclear^1,2^. The creation of various embryonic stem cells (ESCs) and extraembryonic stem cells offers a novel perspective and an exceptional opportunity for in-depth investigation of this developmental window in vitro. Researchers have utilized stem cells from different sources and states to construct various in vitro embryonic models^3–6^. These models, formed through assembly or induction, are designed to mirror distinct phases of in vivo development, offering us entirely new insights into early embryonic development.

Pluripotency—the capacity of a single cell to self-renew and generate all cell types in an adult body, including the three germ layers and their derivatives—plays a crucial role in development. In vivo, embryonic pluripotency is transient and occurs only during the early stages of embryogenesis as a continuous process. In vitro, there are two primary sources of pluripotent stem cells (PSCs). The first source, embryonic-derived PSCs, is isolated from blastocysts under specific culture conditions^7,8^. The second source involves somatic reprogramming techniques, including transcription factor-mediated reprogramming^9^, chemical reprogramming^10^, and others^11–13^. In accordance with in vivo developmental process, PSCs display a diverse range of states^14^, including naive, primed, and formative pluripotent states. Numerous other states of PSCs also provide exceptional resources for investigating embryonic development and exploring applications in the field of regenerative medicine.

In addition, extraembryonic tissues play an indispensable role in embryonic development by serving as a potent source of inductive signals^15–19^, mediating implantation^20–22^, and providing crucial late-stage nutrition^23,24^. In recent years, several research groups have reported the acquisition of diverse extraembryonic cell lines such as trophoblast stem cells (TSCs) in humans and mice^25–32^, extraembryonic endoderm cells (XENs) during mouse embryogenesis^33–35^, hypoblast cells in humans^36,37^, extraembryonic mesoderm-like cells^38^, as well as amnion-like cells^31,39^. These findings provide valuable models for investigating the development of extraembryonic tissues and maternal–fetal interactions^40,41^.

The precise events and regulatory mechanisms of early embryo development remain largely enigmatic, presenting a significant challenge to scientists^42^. In recent years, researchers have been using ESCs and extraembryonic stem cells to construct in vitro embryo models to study early embryonic development^3–6^. In vitro blastoid formation mimics the process of blastocyst formation and early cell fate determination in vivo, providing an opportunity for the study of infertility and early pregnancy loss^43,44^. The establishment of a gastrula model can simulate key developmental biological events, such as gastrulation^4,45,46^, the formation of amniotic cavity^47,48^, and yolk sac^49,50^, which can provide new insights into embryonic development at the gastrula stage, and can be used for high-throughput genetic and chemical screening. Such embryonic models have substantial research value and application potential, providing insights into the early stages of embryogenesis that were previously unknown.

In this review, we offer a comprehensive overview of our current understanding of mouse and human ESCs, as well as extraembryonic stem cells and their respective culture conditions. In addition, we integrate recent advancements in stem cell-derived mouse and human embryonic models from the angle of distinct initiating stem cell types used in these models. Our primary emphasis is on elucidating these cutting-edge breakthroughs.

## Embryonic stem cells with distinct pluripotent states

**Fig. 1:**
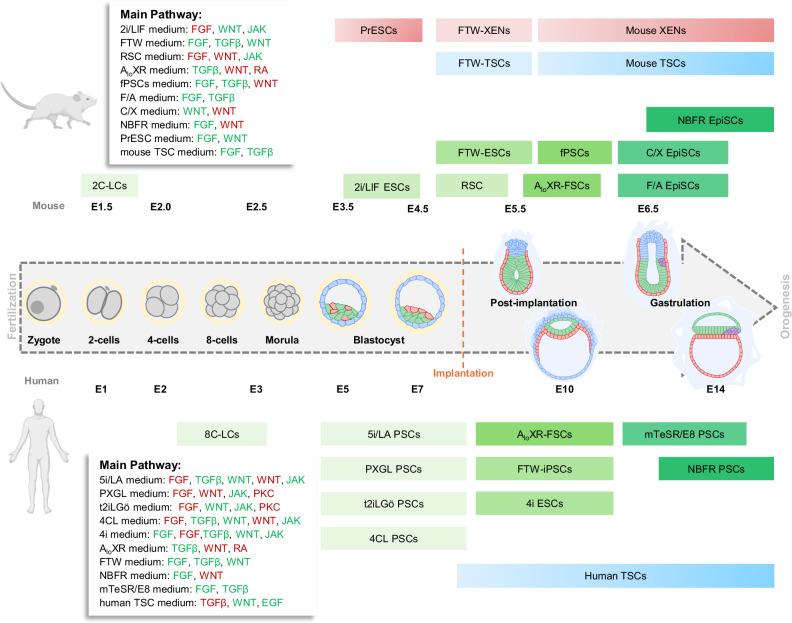
A schematic diagram of the relationship between cell states in different culture systems and their correspondence to in vivo development in humans and mice. The timeline is segmented into three key phases: fertilization, pre-implantation, and post-implantation, indicating specific embryonic days (E) for each species. The upper part of the axis corresponds to mouse development, from fertilization up to E6.5, while the lower part corresponds to human development, from fertilization up to E14. Along the timeline, various stem cell types, primarily ESCs, are listed with their respective culture systems and developmental periods. The periods when embryonic tissues such as TSCs and XENs appear are also correlated with their in vivo counterparts. Different colors signify different stem cell types or conditions. For instance, green shades represent various PSCs, while blue and red shades indicate extraembryonic stem cells such as TSCs and XENs. The gray arrowed area denotes the process from fertilization to gastrulation. Red, pathway inhibition. Green, pathway activation. 2C-LCs 2-cell-like cells, PrESCs primitive endoderm stem cells, FTW-XENs extraembryonic endoderm stem cells cultured in the medium with FGF2, Activin A and CHIR99021, FTW-TSCs trophectoderm stem cells cultured in the medium with FGF2, Activin A and CHIR99021; XENs extraembryonic endoderm stem cells, TSCs trophectoderm stem cells, 2i/LIF ESCs embryonic stem cells cultured in the medium with MEK inhibitor PD0325901, the GSK3 inhibitor CHIR99021, and LIF, RSC rosette-like stem cells, A_lo_XR-FSCs formative stem cells cultured in the medium with a relatively low concentration of Activin A, the WNT inhibitor XAV939, and the retinoic acid antagonist BMS493, F/A EpiSCs epiblast-derived stem cells cultured in the medium with FGF2 and Activin A, NBFR EpiSCs epiblast-derived stem cells cultured in the N2B27 basal medium containing FGF2 and IWR-1, FTW-ESCs embryonic stem cells cultured in the medium with FGF2, Activin A, and CHIR99021, fPSCs formative state pluripotent stem cells cultured in the medium with FGF2, Activin A, and XAV939, C/X EpiSCs epiblast-derived stem cells cultured in CHIR99021/XAV939 or CHIR99021/IWR-1 condition, 8C-LCs 8-cell-like cells cultured in enhanced medium with higher concentrations of DZNep and TSA, 5i/LA PSCs pluripotent stem cells cultured in five inhibitors medium with LIF, PXGL PSCs: PSCs cultured in the medium with PD0325901, XAV939, Gö6983, and LIF, t2iLGö PSCs PCSs cultured in two inhibitors medium with Gö6983, 4CL PSCs PSCs cultured in the medium with PD0325901, CHIR99021, IWR-1, Activin A, LIF, and two epigenetic factors, A_lo_XR-FSCs formative stem cells cultured in the medium with a relatively low concentration of Activin A, the WNT inhibitor XAV939, and the retinoic acid antagonist BMS493, FTW-iPSCs induced pluripotent stem cells cultured in the medium with CHIR99021, Activin A, FGF2, 4i ESCs embryonic stem cells cultured in four inhibitors medium with LIF, FGF2, and TGFβ, mTeSR PSCs PSCs cultured in mTeSR medium, NBFR ESCs cultured in the N2B27 basal medium with IWR-1, FGF2.

ESCs are the in vitro counterpart to the epiblast of early embryos, representing a very early stage of embryonic development. ESCs have two unique features that provide them with great potential for utility in regenerative therapies and also for studying early embryonic development. First, different from the transient emergency of pluripotency in embryos, ESCs have the ability of self-renewal, which can be maintained indefinitely as a pure population of pluripotent cells in culture, offering us a powerful tool for researching the internal development process and the orchestration of the whole body. Second, ESCs have the potential to differentiate into any tissue of an adult organism, which serves as “seed cells” that enable the achievement of organ regeneration. This gives ESCs great potential for regenerative medicine and disease researches, providing a possible material for clinical organ transplantation and also opening up possibilities for stem cell therapy. Over the past few decades, various protocols and culture conditions have been established to enable the derivation of a broad spectrum of ESCs^51^. Since pluripotency in vivo exists on a continuous spectrum, plenty of pluripotent states have been identified, ranging from naive to primed, including intermediate transition states like the formative state. In addition, some ESCs exhibit unique differentiation abilities, such as totipotent-like stem cells^51^. Here, we will focus on the established cell lines and the culture conditions that stabilize these cells at distinct pluripotency states (Fig. 1).

### Mouse naive pluripotency

Naive ESCs are the in vitro representation of epiblast in pre-implantation blastocysts (Fig. 1). In 1981, ESCs were successfully derived by co-culturing isolated mouse epiblast with mouse embryonic fibroblasts (MEFs) in a condition containing serum (10% fetal calf serum and 10% newborn calf serum, or only with 10% calf serum)^7,52^. The following studies have led to the progressive improvement of ESC culture conditions^53–56^. Currently, the commonly used condition for the maintenance of mouse ESCs is the serum-free 2i/LIF condition, which includes the MEK inhibitor PD0325901, the GSK3 inhibitor CHIR99021, and LIF^57,58^. Remarkably, it is widely accepted that all established mouse ESCs can be propagated under this condition^59^. In addition, mouse naive cells, including those cultured in 2i/LIF condition, are capable of forming high-efficiency blastocyst chimeras^60^. However, the long-term culture of mouse ESCs in 2i/LIF condition has been shown to result in genetic and epigenetic aberrations and impair developmental potential, attributed to the MEK inhibitor PD0325901^61^. Mechanistically, the primary mechanism of these compounds is the inhibition of MEK1/2, which is partially accomplished through the downregulation of DNA methyltransferases and their associated cofactors^61,62^.

### Mouse primed pluripotency

Twenty-six years after mouse ESCs were stabilized from pre-implantation epiblast, another type of pluripotent embryonic stem cell was derived from the mouse post-implantation epiblast. These cells were successfully stabilized in vitro using a new culture condition containing Fibroblast Growth Factor 2 (FGF2) and Activin A, referred to as the FA condition^63,64^. These newly derived cells were named epiblast-derived stem cells (EpiSCs). Following the establishment of both ESCs and EpiSCs, researchers realized that distinct pluripotent states exist. While mouse ESCs represent the “naive” state of pluripotency, the newly derived EpiSCs represent “primed” state of pluripotency. The specific features of the naive and primed states have been well summarized in previous reviews^14,59,65,66^. Later, by stabilizing β-catenin through a combined action of GSK3 inhibitor (CHIR99021) and tankyrase inhibitor (XAV939 or IWR-1)^67–69^, EpiSCs maintained under this condition are more closely related to ESCs in development than EpiSCs maintained under FA condition. This is known as the CHIR99021/XAV939 or CHIR99021/IWR-1 condition^67^. Following this, another alternative type of mouse EpiSCs using a condition containing FGF2 and IWR-1 (dubbed the NBFR condition) was established^70^. Interestingly, EpiSCs in the NBFR condition uniquely represent cells of the posterior-proximal epiblast of mouse post-implantation embryos^70^ (Fig. 1). In contrast, EpiSCs in the classic FA condition transcriptionally correspond to the region of the late-gastrula anterior primitive streak^71^.

### Mouse formative pluripotency

In 2017, Austin Smith’s team hypothesized the existence of a third, intermediate pluripotent state, positioned between the naive and primed states as part of a developmental continuum (Fig. 1). They called this third state of pluripotency the “formative state”^72^. These cells were thought to correspond to the E5 to E6 pre-streak epiblast that arises just before gastrulation and has the unique ability to respond directly to primordial germ cell (PGC) induction cues^72^. A few years earlier, an important study by Saitou and colleagues reported a unique epiblast-like cell (EpiLCs) type that could be generated transiently by culturing naive mouse ESCs in the FA condition. Interestingly, EpiLCs demonstrated direct responsiveness to PGC induction: a key feature of formative pluripotency^73^. However, EpiLCs could not be maintained stably in culture and thus only exist as a transient cell type. However, three independent groups have recently reported the generation of stable ESCs that resemble the formative state. Smith and colleagues derived formative stem cells using a relatively low concentration of Activin A, the WNT inhibitor XAV939, and the retinoic acid antagonist BMS493 (called the A_lo_XR condition)^74^. The formative ESCs generated by Wu and colleagues (called FTW-ESCs) were maintained in an alternative condition that contains FGF2, transforming growth factor β (TGF-β)/Activin A, and the GSK3 inhibitor CHIR99021^75^. The third report on formative ESCs by Li and colleagues used a Matrigel-based 3D culture condition that combines FGF2, Activin A, and inhibition of the WNT pathway by XAV939. The resulting formative cells (referred to as fPSCs) could be stably maintained as 3D embryoids^76^. fPSCs undergo morphological changes by switching to a polarized epithelial state, display transcriptomic and epigenomic features distinct from those of naive and primed ESCs, which are transcriptionally similar to E5–E6 epiblast. Importantly, fPSCs can be directly and efficiently induced into PGCs. In the culture conditions with FGF2, Activin A, and XAV939, the epithelial 3D structure can be maintained, but there are certain issues with long-term culture. The Shahbazi lab’s research improved the culture conditions by adding Noggin, which better inhibited the activity of BMP signaling pathway, thereby maintaining the epithelial structure and the expression of pluripotent genes^77^.

### Mouse 2-cell-like state

The ability of a single cell to form an entire organism is referred to as totipotency^78,79^. In mice, only zygotes and blastomeres of 2-cell embryos are totipotent^80^. In 2012, Macfarlan and colleagues identified a subpopulation of cells within naive mouse ESCs cultures that resemble the 2-cell blastomere. These were named 2-cell-like cells (2C-LCs)^81^. The 2C-LCs share several key features with 2-cell embryos, including the ability to develop into extraembryonic lineages. Several subsequent studies have identified regulating factors that could promote the reprogramming of naive mouse ESCs into 2C-LCs, such as double homeobox (DUX) and long interspersed nuclear element-1 (LINE1)^82–85^. Similar to the aforementioned EpiLCs, 2C-LCs are a transient cell type that could not be stably maintained in culture. Recently, by inhibiting RNA splicing using the inhibitor Pladienolide B (PlaB) in the serum-LIF condition, Du and colleagues successfully derived stable totipotent blastomere-like cells (TBLCs) that could be cultured long-term. TBLCs share some molecular features with 2-cell and 4-cell stage embryos^86^. Another study by Wang and colleagues reported the generation of stable totipotent-like stem cells (TLSCs), which were shown to be the most accurate in vitro representation of mouse 2-cell embryos to date^87^. Importantly, both TBLCs and TLSCs demonstrated a robust bidirectional developmental capacity to generate embryonic and extraembryonic lineages^86,87^. However, it has not yet been verified whether these cells can generate an organism from a single cell, in the strict sense of totipotency.

### Other alternative mouse cell types

In addition to the pluripotent and totipotent states described above, several other unique mouse stem cells have been established^88–91^, each possessing specific characteristics that distinguish them from traditional pluripotent and totipotent cells. As there is no consensus^92^ in the field on how to classify these alternative cell types, in this review we summarize them in separate section^88–91,93–96^. In 2017, the groups of Deng and Liu extended or “expanded” the developmental potential of mouse PSCs toward totipotency, generating a unique cell type called expanded pluripotent stem cells (EPSCs)^88,89^, accomplished this through a condition known as LCDM with LIF, the GSK3 inhibitor CHIR99021, (S)-(+)-dimethindene maleate (DiM) and minocycline hydrochloride (MiH). EPSCs displayed certain totipotent characteristics, such as the upregulated expression of totipotency-associated genes and the ability to give rise to both embryonic and extraembryonic tissues when injected into a mouse embryo^88,89^. However, recent studies have dismissed the possibility that EPSCs are bona fide totipotent^95,97^, a topic that remains under significant debate. Although it has been claimed that the contribution of EPSCs to the trophectoderm (TE) lineage remains to be investigated, recent findings demonstrated that EPSCs do not contribute to the TE. In addition, the state of EPSCs is not consistent with the early cleavage state^95^. Another alternative cell type that does not quite fit traditional criteria is rosette-like stem cells (RSCs), derived by Berge and colleagues. Through the addition of the WNT inhibitor IWP2 and the ERK inhibitor PD0325901, but in the presence of LIF (called LIM condition), RSCs express naive pluripotency markers while exhibiting some features of formative pluripotency, such as the upregulated expression of formative pluripotency markers and a polarized epithelial morphology^91^. However, the responsiveness of RSCs to PGC induction cues, a key feature of the formative pluripotency, has not been investigated, hence the unclear classification of RSCs. Advanced pluripotent stem cells (ASCs), derived by Surani and colleagues using Activin A, BMP4, LIF, and CHIR99021 to promote WNT signaling (called ABCL condition), exhibit a transcriptomic profile distinct from naive or primed pluripotent cells, with a stable hypermethylated epigenome^90^. ASCs are developmentally less advanced than naive PSCs, but has greater potential than primed pluripotent stem cells, which leads them to develop more strongly in embryo, PGC, and yolk sac of the chimera (Table 1).

**Table 1:**
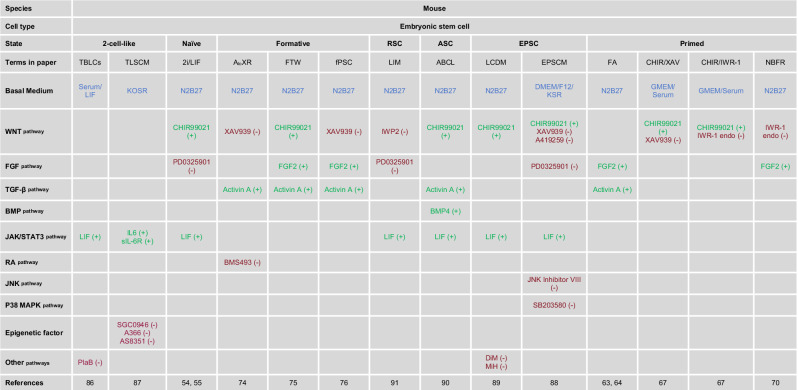
The culture conditions of the different mouse embryonic stem cells.

Red (−), pathway inhibition. Green (+), pathway activation. Blue, basal medium.

Molecule compounds function: CHIR99021: an inhibitor of the GSK3; XAV939: a tankyrase inhibitor; IWR-1 endo: a tankyrase inhibitor; IWP2: a WNT pathway inhibitor; inhibits Porcupine; PD0325901: a MEK inhibitor; LIF: Leukemia Inhibitory Factor, the activator of the JAK kinase family; BMS493: an inverse agonist for retinoic acid receptors; JNK Inhibitor VIII: a JNKs (which are a group of MAPKs) inhibitor; SB203580: an inhibitor of p38 MAP kinase; SGC0946: a methyltransferase DOT1L inhibitor; A-366: a methyltransferase G9a/GLP inhibitor; AS8351: an inhibitor of KDM5B; MiH: minocycline hydrochloride, a PARP inhibitor; DiM: (S)- (+)-dimethindene maleate, an inhibitor of the histamine and the muscarinic receptors; PlaB: Pladienolide B, a RNA splicing inhibitor.

### Human primed pluripotency

Before primed mouse pluripotent cells were derived from the epiblast of post-implantation mouse embryos in the form of EpiSCs, human primed pluripotent cells had already been described. Due to ethical reasons and legal limitations, no attempt has been made to derive primed pluripotent cells from human post-implantation embryos. However, human ESCs have been derived from the inner cell mass (ICM) of human pre-implantation embryos^8,98,99^. Although human ESCs and naive mouse ESCs are both derived from the ICM of pre-implantation blastocysts, human ESCs are considered to be in the primed pluripotent state as they demonstrate several noticeable differences from mouse naive ESCs, including conventional human ESCs showing a more flattened shape, low expression of naive pluripotency genes (such as KLF17 and DPPA3), global DNA hypermethylation, H3K27me3 deposition in development-related genes, X-chromosome silencing in female PSCs, as well as loss of pluripotency caused by MEK inhibitor treatment^14,59,65,66^. In addition, human ESCs are dependent on FGF2 and TGFβ1/Activin A signaling for culture, like primed mouse EpiSCs. Current widely used commercial media for human PSC culture (such as mTeSR^99^ and E8 medium^100^) were adapted from the FGF2 and TGFβ1/Activin A culture condition. However, it is worth noting that primed human ESCs, much like primed mouse EpiSCs, can also be maintained in other alternative culture conditions, such as the CDF12 medium containing FGF2 on MEFs^70^. Human ESCs can also be cultured in the NBFR condition (the same condition that can support mouse EpiSCs culture)^70^. Interestingly, vitamin C and l-Proline can regulate the pluripotency of embryonic stem cells by influencing global DNA methylation, transcriptional profiles and energy metabolism. Vitamin C promotes naive states, and l-Proline induces primed pluripotency. It can also capture stable cell lines in an intermediate state between naive and primed^101^.

### Human naive pluripotency

Nearly 30 years after the derivation of naive mouse ESCs, some studies described human ESCs with features resembling naive pluripotency^102–104^. Unlike mouse ESCs, human ESCs could not be maintained under the 2i/LIF condition. However, they could be stabilized in 2i/LIF if the cells were engineered to constitutively express KLF2, KLF4, and OCT4^102^. Since then, several subsequent studies have claimed to generate stable transgene-free naive-like human PSCs^103,105–109^. It is important to note that the capture of human naive cells is a gradual process that is not yet complete, and whether these naive-like ESCs truly represent the in vitro counterparts to the human ICM (as naive mouse ESCs do to the mouse ICM) remains unclear^14,66^. Understanding the similarities and differences between human naive cells and the ICM is crucial for using these cells to create embryo models. Current evidence suggests that while human naive cells share some characteristics with the ICM, such as specific gene expression profiles and the ability to differentiate into multiple lineages, there are notable differences as well. For instance, human naive cells may not fully recapitulate the epigenetic state of the ICM and may exhibit variations in signaling pathway activity. These discrepancies highlight the need for further research to determine the how closely naive stem cells mirror their in vivo counterparts, which is essential for an accurate modeling of human embryonic development^103,107,110^. However, Jennifer Nichols and Austin Smith lab demonstrated that human naive cells could be directly derived from human embryos^111^. By dissociating ICM to isolate epiblast and primitive endoderm (PrE), stem cell colonies emerged directly when exposed to inhibitors of MAPK/ERK, GSK3, and PKC. This suggests that previous culture conditions may have been insufficient to maintain naive pluripotency, likely due to developmental signals from extraembryonic endoderm^112^ in ICM explants or the effects of FGF and serum factors. At the time of writing, three predominate culture conditions are used for the derivation of human naive-like PSCs and for the conversion of primed human PSCs to the naive state. The naive condition t2iLGö is from the Smith lab, which contains the GSK3 inhibitor CHIR99021, the ERK inhibitor PD0325901, the PKC inhibitor Gö6983, and LIF^103^. The 5i/LA condition is developed by the Jaenisch lab, which utilizes five kinase inhibitors, LIF, and Activin A^107^. The third condition, from the Smith lab, is known as the PXGL condition. This condition, like t2iLGö, contains PD0325901, Gö6983, and LIF, but contains the tankyrase inhibitor XAV939 instead of CHIR99021^103,113^. Besides these three conditions, a commercially available naive human PSCs media called RSeT, which was adapted from the Hanna lab's naive human stem cell medium (NHSM)^106^, is also commonly used, though this condition has been reported to support a later stage of development^114^. In 2022, researchers added PD0325901, IWR-1, Activin A, Vitamin C, and LIF to the N2B27 medium to culture cells, naming this medium 4CL^115^. These colonies exhibited a morphology similarity to naive PSC colonies and demonstrated the highest levels of naive pluripotency gene expression. The 4CL medium supports long-term maintenance (tested up to 15 passages) without significant karyotype abnormalities. The removal of vitamin C reduced the expression of naive pluripotency genes during the 4CL-mediated conversion process. In addition, RNA sequencing confirmed that PSCs grown in 4CL exhibited transcriptional similarities to early ICM^115^. These systems are also applied to other primates in addition to humans. In the PXGL system, primate ESCs cannot be stably passaged beyond five times. Also, it was found that primate cells in the 4CL and 5i/LA systems induce higher levels of naive marker gene expression^116^. In addition, rhesus monkey naive induced pluripotent stem cells (iPSCs) established under the 4i system (comprising CHIR99021, PD0325901, SP600125, and SB203580) exhibit gene expression characteristics similar to those of mouse and human naive PSCs. This includes the upregulation of key genes such as NANOG and PRDM14, which is crucial for maintaining pluripotency and inhibiting lineage differentiation^117^. The generation of naive iPSCs in primates indicates that naive and primed pluripotent states are conserved across species.

It is worth noting that chromosomal instability of cultured human naive-like PSCs is a great concern in the field^107,110,118^. For example, after 20 passages in 5i/LA culture medium, the cells showed general karyotype abnormalities, with some cell lines showing up to 70% chance of polyploid by passage 15. In contrast, chromosomal instabilities were notably reduced under other culture conditions such as NHSM and RSeT^118,119^. These chromosomal abnormalities could be caused by the presence of the MEK inhibitor PD0325901, which was previously shown to cause the erosion of genomic imprinting and compromise developmental potential^61,62^. Recently, to address the issue of genomic instability, several optimized human naive PSC conditions have been developed, either by titration^120^ or replacement^30,121^ of the PD0325901.

### Human formative pluripotency

Distinct from mouse primed PSCs, human PSCs in the primed state can be directly induced to become PGCs^122,123^, though this is a key feature of the formative state^72^. The ability of human primed PSCs to give rise to PGCs may also be related to differences in the timing and origins of PGCs in primate and mouse. Human primed PSCs, theoretically, should not have this ability. However, this developmental contradiction may be due to cell heterogeneity, cell plasticity, or the late appearance of cells with PGC differentiation capability in human epiblast^124^. Recently, two groups have described conditions that allow for the stable culture of human PSCs resembling the formative state. Wu and colleagues generated an intermediate type of human PSCs that were between the naive and primed states using the FTW condition (the same condition previously mentioned for maintaining formative mouse ESCs)^75^. Human PSCs in the FTW condition demonstrated transcriptional similarity to the E8 human epiblast and displayed the ability to differentiate into PGCs. At the same time, Smith and colleagues reported the derivation of human formative-like cells from both human embryos and naive ESCs using the A_lo_XR condition (the same condition previously mentioned to culture formative mouse PSCs) (Fig. 1). Human PSCs cultured in this condition exhibited a transcriptional profile similar to the monkey post-implantation epiblast^74^.

### Human 8-cell-like state

As previously mentioned, 2C-LCs arose at a very low frequency in mouse naive ESCs cultures^81^. Using a conceptually similar strategy, two studies have captured human cells resembling totipotent-like 8-cell-stage human embryos (called 8C-LCs) in human naive ESCs cultures^125,126^. The report on human 8C-LCs comes from the Reik lab, where they found that in human naive PSCs cultures (i.e., using the 5i/LA, t2iLGö, and PXGL conditions), a small subpopulation of cells (~1.5%) expresses markers of zygotic genome activation (ZGA, refer to refs. ^127–129^) and is transcriptionally similar to human 8-cell stage embryos^125^. Included in this transcriptional similarity is the expression of 8-cell-stage embryo transposable elements in 8C-LCs.

A short time later, an independent study from the Aranguren lab reported that ~1.7% of human cells in the 5i/LA condition exhibit upregulation of markers associated with human 8-cell stage embryos and downregulation of pluripotency-associated markers^126^. Further supporting these findings, Wang lab demonstrated that distinct cell populations in naive human PSCs cultures closely resemble the totipotent-like 8-cell stage of human embryos, highlighting the presence of cells analogous to TE and PrE^130^. Similarly, Aranguren lab provided evidence of early human embryo-like cell populations within naive human PSCs cultures, emphasizing their transcriptional and cellular resemblance to 8-cell stage embryos^126^. Recently, Esteban and colleagues reported a rapid and relatively efficient method for isolating human 8C-LCs starting from primed and naive PSCs^115^. However, 8C-LCs are not stable and cannot be passaged multiple times in vitro. These studies open the doors for studying human totipotency and ZGA programs in vitro. Currently, human totipotent cells with the highest developmental potential cannot be stably cultured in vitro.

In 2024, the Du Lab^131^ conducted a transcriptome analysis on primed hESCs after 48 h of treatment with the splicing inhibitor Pladienolide B (PlaB). The results demonstrated a significant downregulation of blastocyst-specific genes, including key primed genes such as PODXL, DNMT3B, and THY1. Concurrently, genes specific to the 8-cell stage, such as GADD45A/B, MED26, and SNAI1, are markedly activated. The overall transcriptome of these cells closely resembled that of in vivo ZGA stage 8-cell embryos. These ZGA-like cells were designated as ZLCs (ZGA-like cells). Interestingly, a novel MYCP medium containing low concentrations of PlaB was developed, enabling the stable culture of these cells while maintaining normal karyotype, proliferation rate, and cell viability. This MYCP medium can stably culture human 2–4-cell stage cells^131^.

Comparative transcriptome analysis between 8C-LCs and ZLCs revealed notable differences. Both cell types are enriched for a set of 8-cell-specific genes, referred to as common ZGA genes, including HIST1H2BG/BK, GADD45A and GPATCH3. However, ZLCs do not express typical ZGA genes like DUXA/B, ZSCAN4/5B, LEUTX, and TPRX1, which are characteristic of 8C-LCs. Instead, ZLCs express a unique set of 8-cell-specific genes, termed ZLC/ZGA genes, such as ZNF23/34, FAM32A, HBEGF, and MED26. Importantly, many typical naive pluripotency genes that remain active in 8C-LCs are not expressed in ZLCs. Further single-cell transcriptome analysis showed that ZLCs uniformly activate ZGA genes and silence pluripotency genes at the single-cell level, closely resembling the in vivo 8-cell stages^131^.

### Other alternative human cell types

In 2017, Deng and colleagues derived a type of PSCs called extended pluripotent stem cells (EPSCs), which have the ability to contribute to both embryonic and extraembryonic lineages in chimera experiments, through a chemical cocktail (LCDM) containing hLIF, the GSK3 inhibitor CHIR99021, (S)-(+)-dimethindene maleate (DiM) and minocycline hydrochloride (MiH). These cells can be generated from blastocysts, converted from primed human PSCs or induced by somatic reprogramming^89^. Two years later, another group Liu also maintained human EPS cells using a different culture medium, under conditions analogous to those of porcine EPS cells, pEPSCsM, which includes the GSK3 inhibitor CHIR99021, the SRC inhibitor A419259, the inhibitor of Tankyrases XAV939 and supplements vitamin C (Vc), Activin A and LIF^132^. Similarly to mouse EPS cells, the state of human EPS cells and their potential totipotency remains controversial. These cells exhibit features of primed pluripotency in mice^95^ and humans^28,97,133^. In addition to the cell lines aforementioned, there are a number of alternative human cell types that have been reported that do not have a clear-cut potency state. Besides, Surani and colleagues established germline-competent PSCs in a condition comprising four inhibitors, LIF, FGF2, and TGFβ1 (named 4i human ESCs)^134^. While 4i human ESCs display the formative pluripotency feature of direct induction to PGCs, other formative features of these cells had not been investigated. Recently, it was reported that metabolic changes in PSCs could drive pluripotency state changes. By depriving human PSCs lipids (using Essential 8 minimum media), Studer and colleagues captured human PSCs in a naive-to-primed intermediate state, which exhibit several naive-like features even in primed-sustaining conditions^135^. By briefly inhibiting mTOR, Feng and colleagues were able to convert human PSCs into a naive-like state using the 2i/LIF condition^136^. Together, these studies indicate that pluripotent state transitions are not simply induced by culture adaptations (Table 2).

**Table 2:**
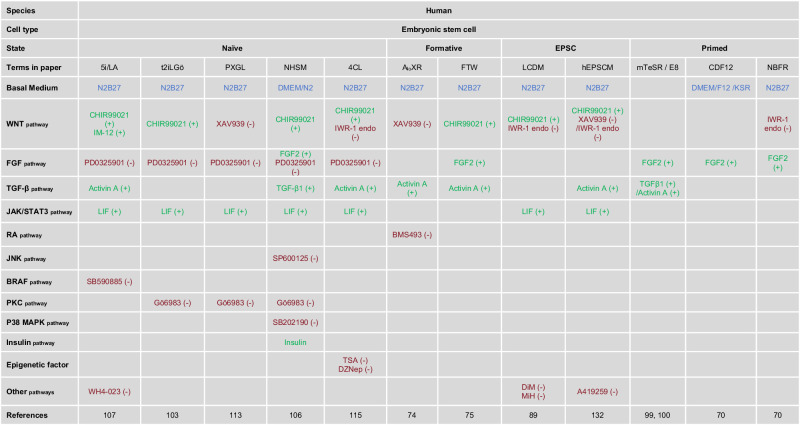
The culture conditions of the different human embryonic stem cells.

Red (−), pathway inhibition. Green (+), pathway activation. Blue, basal medium.

Molecule compounds function: CHIR99021: an inhibitor of GSK3; IM-12: targets GSK3 and activates WNT pathway; WH4-023:an inhibitor of the kinases Lck and Src; XAV939: a tankyrase inhibitor; IWR-1 endo: a tankyrase inhibitor; A419259: a kinase inhibitor with specificity towards Src family kinases; PD0325901: a MEK inhibitor; BMS493: an inverse agonist for retinoic acid receptors; SP600125: a JNK inhibitor; SB590885: a BRAF kinase inhibitor; Gö6983: a broad-spectrum kinase inhibitor; SB202190: a p38 MAP kinase inhibitor; TSA: an inhibitor of histone deacetylase HDAC class I/II; DZNep: an inhibitor of histone methyltransferase EZH2; MiH: minocycline hydrochloride, a PARP inhibitor; DiM: (S)- (+)-dimethindene maleate, an inhibitor of the histamine and the muscarinic receptors.

## Derivation of extraembryonic stem cells

Throughout embryonic development, extraembryonic tissues play critical roles in various developmental processes. Early on, extraembryonic tissues are crucial in regulating embryo patterning. Crosstalk between embryonic and extraembryonic tissues breaks embryo symmetry, building the three definitive axes of the embryo (anterior–posterior, dorsal–ventral, and left–right)^137,138^. Extraembryonic tissues include TE, amnion, extraembryonic mesoderm, PrE, and their derivatives. As ESCs serve as the in vitro representation of the epiblast, several culturable extraembryonic stem cell lines have been established to provide in vitro models for TE and PrE (Table 3).

**Table 3:**
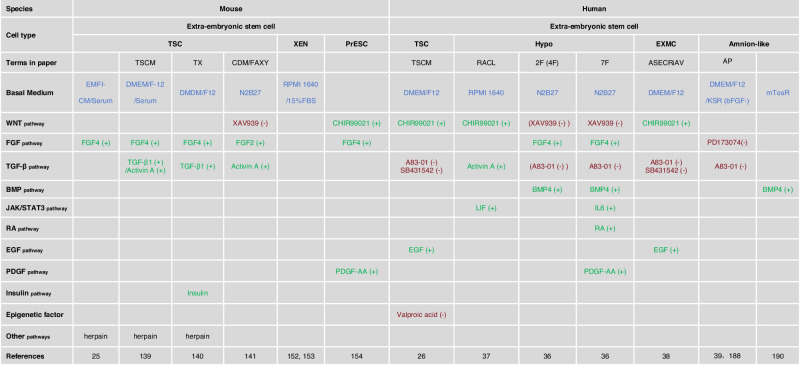
The culture conditions of the extra0embryonic stem cells of both mouse and human.

Red (−), pathway inhibition. Green (+), pathway activation. Blue, basal medium.

Molecule compounds function: CHIR99021: an inhibitor of GSK3; XAV939: a tankyrase inhibitor; A83-01: A inhibitor of the TGF-β type I receptor ALK5 kinase; PD173074: an inhibitor of FGFR; SB431542: an inhibitor of ALK5, ALK4, and ALK7; Valproic acid: an inhibitor of histone deacetylase HDAC.

### Mouse TSCs

In 1998, the stable in vitro counterpart to trophoblast was derived in the form of mouse TSCs by Rossant and colleagues. They utilized a MEF-conditioned medium supplemented with mouse serum and FGF4^25^. Upon injection into mouse embryos, the TSCs can develop into all cell types of the definitive placenta, indicating their bona fide extraembryonic developmental potential. A few years later, Glimcher and colleagues identified that TGFβ1 and/or Activin A are the key factors present in the MEF-conditioned medium necessary for TSC culture^139^. In 2014, two chemically defined conditions were developed for TSCs culture. The Schorle lab reported the TX condition, which consists of ten chemically defined ingredients including FGF4, TGFβ1, heparin, and insulin^140^. Next, the Ohinata lab reported a distinct medium containing FGF2, Activin A, the tankyrase inhibitor XAV939, and the ROCK inhibitor Y27632^141^. Both conditions allow for the culture of TSCs in a defined feeder-free condition while maintaining the developmental ability to contribute to the mouse placenta. In 2022, Rivron lab described a chemically defined medium that captured in vitro stable and highly self-renewing TSCs resembling the blastocyst stage by preventing the heterogeneous state of TSCs^142^. In 2023, Wu lab established extraembryonic stem cell lines from mouse and cynomolgus monkey blastocysts using FTW medium, enriched with activators of FGF, TGF-β, and WNT pathways^143^.

Besides deriving TSCs from mouse embryos, TSCs can also be reprogrammed from naive mouse ESCs by transgenic expression of trophoblast-lineage-determining genes, such as CDX2, EOMES, GATA3, ELF5, TEAD4, ETS2, and TFAP2C^144–151^. Some of these ESCs-derived TSCs even demonstrated all of the developmental capabilities of conventional embryo-derived TSCs^144,146,150^.

### Mouse XENs

The generation of stem cells that represent the third lineage of the mouse blastocyst, XENs, was pioneered by the Rossant lab^152^ using culture conditions based on the protocols used for deriving TSCs and ESCs. The XENs established can be maintained in serum without additional factors and the MEF feeders^152,153^. However, these XENs do not fully recapitulate the developmental potential of the PrE^152^. Recently, Ohinata and colleagues reported the derivation of mouse primitive endoderm stem cells (PrESCs) using a condition containing FGF4, heparin, platelet-derived growth factor (PDGF-AA), and a high concentration of CHIR99021 with MEFs^154^. PrESCs were reported to recapitulate properties of E4.5 PrE and can generate functional PrE-derived tissues. Similar to what was seen in ESC-derived TSCs, XENs can also be generated from naive mouse ESCs by constitutive expression of PrE-specific genes, such as GATA4, GATA6^155–157^ or SOX17^158^. Two groups have also established methods to generate ESC-derived XENs by simple culture adaptation^34,159^. Wu lab discovered that isolating XENs from mice can also establish cell lines in a culture system that activates FGF, TGF-β, and WNT signaling pathways^143^. However, till now, a chemically defined condition for XEN derivation and culture has remained elusive.

### Human TSCs

Stable human TSCs were not derived until 2018, when Okae and colleagues reported the derivation of human TSCs from human villous cytotrophoblast and blastocysts^26^. They utilized a condition containing WNT activator, TGFβ inhibitor, inhibition of histone deacetylase, EGF, and Y27632^26^. These human TSCs have the capacity to give rise to columnar trophoblast identity independently of the tissue of origin in vitro^160,161^ and show transcriptional similarity to in vivo isolated tissues. Interestingly, unlike mouse TSCs which require transgene expression to be converted from naive mouse cells, human TSCs could be rapidly and efficiently converted from naive human PSCs by simple culture adaptation via ERK inhibition and Nodal inhibition^27,31,32^. Consistent with this finding, epiblast cells of the human blastocyst had also been shown to give rise to TE^31^, suggesting that species-specific plasticity is retained in human pre-implantation lineages. However, there are some controversies, as this conversion is very inefficient, which might be due to the presence of residual naive cells in the primed culture. In addition to TSC derivation from human naive cells, recent studies have shown that human TSCs could also be derived from primed human PSCs^162–168^. Though primed-derived human TSCs demonstrate full developmental potential, their real identity is still under debate as independent studies suggested that human TSCs derived from primed PSCs do not meet all trophoblast criteria and that the cells may be an amniotic cell, rather than true TSCs^31,32,169^. Also, some groups have reported the generation of TSCs through reprogramming, in which the cells were maintained in a defined culture medium^170–172^.

### Human hypoblast stem cells

In 2019, Brickman and colleagues captured a cell type resembling human early hypoblast, which they named naive extraembryonic endoderm (nEnd)^37^. They did this by exposing human naive PSCs to a high concentration of Activin A, CHIR99021, and LIF. The nEnd cells produce hypoblast-related basement membrane components and can be differentiated into visceral endoderm. Moreover, these cells share transcriptional features with the in vivo hypoblast^97^ of pre-implantation primate embryos. Nevertheless, the proliferation of nEnd cells cannot be maintained long-term^37^. The Zernicka-Goetz lab established conditions to differentiate hPSCs into yolk sac-like cells (YSLCs) that resemble the post-implantation human hypoblast both molecularly and functionally. YSLCs show the expression of pluripotency and anterior ectoderm markers at the expense of mesoderm and endoderm markers^173^. Recent studies have demonstrated that the addition of BMP4 and FGF4 to the culture media can effectively induce human hypoblast lineage cells from naive human PSCs under specific conditions^36^. This highlighted a significant advance in our understanding of early human development. Unlike in mice^34,174^, the specification of human hypoblast does not require retinoic acid and PDGF, suggesting unique aspects of human embryogenesis. Furthermore, the induced hypoblast-like cells closely resemble the pre-implantation, blastocyst-stage hypoblast, which provide a valuable model for studying the early stages of human development and the role of the hypoblast in supporting epiblast development^36^. To date, direct derivation of stable hypoblast stem cells from human blastocysts and stable cultured authentic human hypoblast stem cells has not yet been achieved.

### Human extraembryonic mesoderm cells (EXMCs)

In early embryonic development, EXMCs are an important class of extraembryonic tissue cell types. In rodents, EXMCs originate from gastrulation^175,176^, whereas primate EXMCs not only appear after gastrulation but also appear before gastrulation^177,178^, whose origins are the subject under many debates^178–183^. There is little research available on the culturing of EXMCs at present. In 2022, Vincent Pasque’s team^38^ have induced naive stem cells to EXMCs by inhibiting Nodal and GSK3B and activating the mTOR and BMP4 signaling pathways, and found that EXMCs originate from a group of intermediate epiblast cells. In 2023, Li’s team^184^ also induced XEN-like cells and EXMC-like cells by activating WNT and BMP4, meanwhile additional inhibition of the Nodal and Activin signaling pathways resulted in only EXMCs. Both studies showed that the EXMCs were induced by activating BMP4 and inhibiting Nodal signaling pathway. In the post-implantation embryo model^3^, Activin A was withdrawn from the RACL culture system. Subsequently, a certain proportion of EXMCs was present in the PrE culture system. However, the origin of these EXMCs remains unknown. Some evidence also exists that a portion of EXMCs originates from trophoblast and PrE in vivo^178,181,185^, but further experimental validations are still needed.

### Human amnion-like cells

Amnion cells, a type of extraembryonic cell, emerge from the pluripotent epiblast around the time of implantation. This is followed by epithelialization and cavitation, forming the amniotic sac, which occurs before gastrulation^186^. The diversification of amniogenesis mechanisms remains unclear.

In 2017, the Fu lab reported in vitro induction of amnion-like epithelial structures using BMP4 treatment on 3D-cell aggregates^47,48^. In 2021, the Austin Smith lab discovered that naive cells can be induced into trophoblast cells in response to inhibition of ERK and Nodal signaling, while primed cells respond to BMP4 by inducing amnion-like cells^31^. The previous research indicated that inhibition of SMAD2/3 is accompanied by the increase in SMAD1/5/8 signaling, suggesting a negative feedback loop in which suppressing one pathway enhances the activity of the other^187^. It is hypothesized that SMAD1/5/8 activation in primed cells may further stimulate amnion marker expression.

In 2022, Eiraku lab revealed that chemical blockade of Activin/Nodal and FGF signals activates BMP signaling, steering human primed ESCs into GATA3-expressing cells with phenotypes resembling syncytiotrophoblasts and amnion ectoderm^39^. Single-cell analysis by the Rugg-Gunn lab identified two distinct waves of amnion formation in primates. Partially primed hPSCs temporarily differentiate into early amnion-like cells, while fully primed hPSCs produce cells akin to late amnion. This dual wave formation suggests that inhibition of SMAD2/3 and activation of SMAD1/5/8 are crucial for inducing amnion cell markers in primed cells^188^. The role of FGF in BMP4-induced amnion cell differentiation remains unclear. Both inhibition and activation of FGF can yield amnion cell types, but FGF activation also induces mesoderm expression^189^. In 2024, Fu lab found that BMP4 treatment for two days can produce amnion cells, which can induce primed cells to differentiate into PGCs when co-cultured^190^. However, the heterogeneity of amnion cells obtained through current methods remains high.

## Engineering early developmental models by using PSCs

**Fig. 2:**
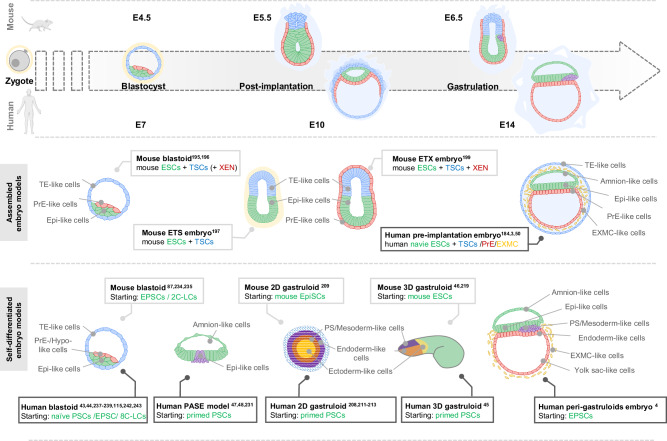
A diagram illustrating the correspondence between developmental stages in mice and humans and their alignment with current embryonic model periods. This detailed diagram illustrates the various stages of early embryonic development in mice and humans, including assembled embryo models and induced embryo models. The timeline at the top marks the embryonic development stages in mice from the zygote to the E14 stage, as well as the corresponding stages in human development. The lower half of the image categorizes two main methods of in vitro emulation. In the “Assembled embryo models” section, different cell types, such as ESCs and TSCs, are combined to simulate the structures and functions of early embryos. In the “Induced embryo models” section, cells differentiate and organize into structures without the guidance of extraembryonic cells. As shown, gastruloid models for both mice and humans self-organize from stem cells and can simulate the gastrulation stage of the embryo. The numbers in the diagram refer to the cited scientific references. TE trophectoderm, PrE primitive endoderm, Epi epiblast, ESC embryonic stem cell, TSC trophoblast stem cell, XEN extraembryonic endoderm, EXMC extraembryonic mesoderm cells, EPSC extended pluripotent stem cell, 2C-LC 2-cell-like cells, EpiSC epiblast stem cell, PSC pluripotent stem cell, PS primitive streak, 8C-LC 8-cell-like cells.

Building on previous stem cell research, we further consider the importance of stem cells in constructing various embryonic models at different developmental stages. The establishment of a broad spectrum of embryonic and extraembryonic stem cell lines has created new opportunities to construct in vitro developmental models that allow us to study the key events regulating embryonic development. Up to now, a range of embryonic models that recapitulate various developmental stages, ranging from pre-implantation to gastrulation, have been developed. Each model is specifically designed to emulate particular aspects of embryonic growth. The unique characteristics and developmental progress of these models have been reviewed previously^1,6,191–193^. We further provide a summary of the starting cell types (or cell “building blocks”) that have been utilized in existing embryo models, and highlight their comparative advantages of different cell types in embryonic model development. Depending on whether or not all cell lineages are present at the start of the embryo model, models can be classified into two types: “assembled” embryo models and “induced” embryo models (Fig. 2). The former involves bringing together distinct cell types to form a cohesive unit that mimics an embryo at a certain stage, while the latter relies on the inherent potential of a single stem cell type to differentiate and organize into a structure resembling a specific stage of embryonic development. These methodologies underscore the versatility and potential of stem cells in providing insights into the early stages of life formation.

### Assembled embryo models

To generate a model that mimics the embryo at a certain developmental stage, it is a natural idea to bring all of individual cell types of the embryo together and allow them to self-organize. This strategy, known as “assembly”, has proven to be very successful (Fig. 2). It is worth noting that cells that combine to form assembled models can and do still undergo differentiation.

#### Mouse

Using this approach, Rivron and colleagues aggregated mouse ESCs and TSCs at a defined ratio and generated the mouse blastocyst-like structure in vitro, called “blastoid”^194^. These mouse blastoids recapitulate the spatial organization and transcriptional profiles of mouse blastocysts^194^. However, post-implantation development of the blastoids is inefficient, which could potentially be explained by the lack of a certain number of PrE-like cells. Later improvements to the protocol involved incorporating PrE cells, the third lineage of the blastocyst, into the blastoids. Rivron and colleagues pre-treated mouse ESCs with a PrE-induction medium, generating 3D PrE-/Epiblast-like cell aggregates, which they then mixed with TSCs to generate advanced mouse blastoids^195^. Another approach, developed by the Zernicka-Goetz lab, was to aggregate mouse TSCs with mouse EPS cells which have been shown to have the potential to develop into PrE, to generate the blastoids^196^. The advanced blastoids generated using both approaches exhibited advanced post-implantation developmental progression and formed egg cylinder morphology in vitro. However, these blastoids formed from TSCs and EPSCs implant at a lower efficiency^142^. In a recent study, Rivron’s lab found that trophectoderm stem cells and ESCs have a stronger ability to form blastoids, not only maintaining the proliferation and self-renewal of localized trophoblasts but also sustaining the secretion of WNT6/7B, which stimulate uterine decidualization^142^. Therefore, these blastoids have a higher implantation efficiency in the uterus. Although the process of decidualization during implantation was observed, the formation of the fetus was not detected.

Another embryo model generated via the assembling approach is ETS/ETX embryo, which resembles mouse gastrula. ETS embryos referred to aggregates of mouse ESCs and TSCs for modeling mouse post-implantation embryonic development rather than the pre-implantation blastocyst^197^. This strategy involves combining single mouse ESCs and small clumps of TSCs, which then spontaneously form aggregates. Through lumenogenesis, fused aggregates form a structure resembling the egg cylinder stage of mouse embryos. Moreover, they recapitulate key events of peri-implantation mouse embryos such as PGC, primitive streak formation and the breaking of anterior–posterior symmetry^197^. This model further demonstrated the importance of incorporating extraembryonic lineages for the development of embryonic cells, especially during the post-implantation period. However, there are some limitations to these models. These studies focused more on the further development of embryonic cells and did not pay sufficient attention to the further development of trophoblast cells during the post-implantation period. In 2018, the Zernicka-Goetz lab made a significant advancement in embryo model research by incorporating mouse XENs into the existing ETS model. This innovation led to the creation of ETX embryos, and in subsequent cultures, the presence of mesoderm and endoderm was discovered^198^, and similar results were obtained in 2019 by the Han lab, who devised a nonadherent-suspension-shaking system to generate self-assembled embryo-like structures (ETX-embryoids)^199^. A recent study found that ETX embryos that incorporate induced XENs (iXEN, cells derived from mouse ESCs transiently expressing GATA4), instead of conventional XENs, showed greater developmental potential^200^.

In 2022, the research teams led by Hanna and Zernicka-Goetz optimized an in vitro culture system for mice^201,202^. They achieved cell types similar to TSCs and XENs by overexpressing genes such as *GATA4* and *CDX2*. These cells were co-cultured with naive cells and maintained in a rotating culture system until they reached a developmental stage close to E8.5 in vivo. This allowed them to recapitulate in vitro key events such as primitive streak formation, hematopoiesis, neurogenesis, heart beating, and organogenesis, utilizing a multi-cell assembly approach.

So far, mouse embryo models are often assembled in vitro using different types of stem cells to form complex embryonic structures. As these models develop, in vitro culture techniques also need continuous development and refinement. The ongoing advancements in the field of embryo model generation highlight the intricate interplay between cellular composition and developmental potential. These innovations, while promising, underscore the complexity of mimicking natural embryogenesis in vitro. Although it is now possible to develop mouse embryos to a stage corresponding to E8.5 in vivo, a deeper understanding of the key developmental events using mouse embryo models is still required.

#### Human

Due to ethical and technical constraints^203–205^, the study of human post-implantation development has long been limited. Until now, there has been a lack of post-implantation human embryo-like models with the proper spatial and morphological features. However, recent researches have further expanded the application of human naive cells in embryo models. By using unmodified human naive ESCs and ESCs overexpressing *GATA4*, *GATA6*, *CDX2*, and *GATA3*, comprehensive human spatially organized morphogenesis (SEMs) was successfully created (Fig. 2)^3^. These SEMs mimic various lineages and regions of post-implantation embryos, including the epiblast, extraembryonic mesoderm, and trophoblast. These human SEMs exhibit characteristics resembling post-implantation embryonic development. It was suggested that only establishing naive hPSC may be sufficient to enable the generation of advanced embryo-like structures in vitro. In recent studies, hypoblast-like cells generated by the medium system (RACL or RCL) resemble those in the post-implantation stage^36,173^. Similarly, naive cells can further differentiate into primed cells, which have the capability to form lumens spontaneously^206^. This may suggest why such an assembly of cells can develop to the post-implantation stage. Therefore, we speculate that the developmental stage and developmental potential of stem cells are crucial for the construction of embryo models.

Furthermore, Zernicka-Goetz lab and Sozen lab have constructed similar embryo models, which resemble early post-implantation embryo development^50,207^. These findings illustrate that endogenous BMP and Nodal are crucial drivers of amnion and primordial germ cell-like cell differentiation from the epiblast-like domain within inducible embryoids. They also revealed functional differences in the gene regulatory networks underlying hypoblast subpopulation differentiation, specifically noting the inhibitory effect of prolonged SOX17 overexpression on CER1-positive anterior hypoblast identity^50^. Since this model cannot implant, it lacks the capacity to develop toward fetal stages and does not replicate stages beyond primitive streak formation or include all cell types of the gastrulation-stage embryo. It is important to note that these embryo models still have limitations and pose several potential challenges that require further refinement in their continued cultivation. Further, researchers created an embryo-like assembly called “E-assembloid” by combining human naive ESCs and extraembryonic cells^184^. They revealed that WNT, BMP and Nodal signaling pathways synergistically orchestrate human peri-implantation lineage development. These models still exhibit certain differences compared to in vivo structures, so it remains to be discussed whether the signaling pathways fully correspond to those in vivo.

Utilizing ESCs in conjunction with cells overexpressing *GATA6*, despite some structural developmental obstacles in 3D, the induction of yolk sac-like cells and amniotic sac structure was achieved through a 2D culture system. Under the influence of GATA6-positive cells, the epiblast developed an anterior–posterior axis and 2D culture system eventually got rise to a large number of early blood cells^49^. Notably, unlike the previously mentioned mouse assemblies^202^, human hypo cells primarily used GATA6 overexpression to indicate the hypoblast, whereas in mice, it was GATA4.

Currently, mouse embryo assembly models, with the aid of in vitro culture systems, can develop to later stages, whereas human models are significantly falling behind. The transition of human embryo models from pre-implantation to post-implantation marks a significant step forward. Although current human assembled embryo models can be cultured in vitro, they cannot be further developed. One of the significant factors is the lack of the correct extraembryonic lineages. They compensate for the absence of extraembryonic cell types by overexpressing extraembryonic tissue genes in stem cells or by inducing extraembryonic cells, which often exhibit high heterogeneity. However, this does not fully mimic the true state of extraembryonic tissues and restricts further development of the embryonic model due to the lack of correct extraembryonic lineage cells.

### Induced embryo models

In contrast to the assembly approach, induced embryo models refer to models generated from a single stem cell type. By taking advantages of the differentiation potential of stem cells, a proper induction method could allow the starting cells to differentiate and self-organize into a model that recapitulates a particular stage of development in vitro.

Due to the limited cross-lineage differentiation capacity of stem cells and the fact that different embryonic lineages use different induction cues, the majority of induced embryo models only partially mimic the development of a single lineage, most commonly epiblast. A prime example of this is the 2D gastrulation model established by Warmflash and colleagues. By treating primed human or mouse PSCs cultured on a circular micropattern with BMP4 (or a WNT activator), the cells undergo a self-organized differentiation process and form radially symmetrical structures that contain concentric circles expressing extraembryonic, endodermal, mesodermal, and ectodermal markers from the outer to inner layers, respectively^208–210^. Despite the fundamental differences between 2D geometry of this in vitro gastrulation model and the 3D topology of in vivo embryos, this 2D gastrulation model provides a defined and reproducible tool for studying dynamic cell signaling-regulated events during early gastrulation^211–218^.

In addition to the 2D gastrulation model, a 3D gastrulation model called gastruloid has been generated solely from mouse ESCs^46,219^ or human ESCs^45^. By transient activation of WNT signaling, mouse or human embryoid bodies (EBs, the aggregates of ESCs) could spontaneously elongate and specify ectoderm-, mesoderm-, and endoderm-like cells in an asymmetric manner, which recapitulates anterior–posterior axial patterning of post-gastrulation mouse and human embryos. Under various induction conditions, EBs can further differentiate into structures such as somites^220–222^, neural tubes^223,224^, trunk-like structures^225,226^, and even a primitive beating heart^227–229^. 3D gastruloids provide an exciting opportunity to study the early stages of mammalian post-implantation development in vitro^230^. Recently, due to the in-depth study of extraembryonic tissues and the limitations of gastruloids, there has been an increase in research on complete embryo models that include extraembryonic tissues.

Another remarkable embryo model generated through the self-differentiation approach is the human post-implantation amniotic sac embryoid (PASE) model, pioneered by the Fu Lab^47,48,231^(Fig. 2). The previous research indicated the formation of lumens is not only a spontaneous process of PSCs but also one that can be controlled by regulating cytoskeletal dynamics^206^. Following previous literatures where spherical epithelia were created by embedding human stem cells in Matrigel^206,232^, the Siggia lab utilized an in vitro 3D model generated from human embryonic stem cells to reveal the molecular mechanisms of axial symmetry breaking in human embryos through BMP4 and WNT signaling^233^. By culturing primed human PSCs on a soft surface coupled with embedding of the cells in an extracellular matrix, Fu and colleagues generated luminal spherical human PSCs colonies and, interestingly, a small fraction of spheres spontaneously forms bipolar structures containing squamous amniotic ectoderm at one end and columnar epiblast at the other. Moreover, these structures resemble the human amniotic sac, both morphologically and transcriptionally^47,48^. To increase the formation efficiency of PASE structures, the Fu team reported an advanced PASE model by taking advantage of microfluidic techniques^231^. By tightly controlling the exposure of human PSCs aggregates to BMP4, the aggregates robustly form amniotic cells on the exposed side and primitive streak-like cells on the opposite side. PASE embryo models represent an embryo model that can recapitulate the development of the amniotic sac compartments during early post-implantation development of the human embryo^191^.

It is important to emphasize that the developmental potential of the starting cell is the determining factor of induced embryo models. Starting with cells that have the capacity to give rise to extraembryonic lineages (i.e., either through totipotency or plasticity, as mentioned in the previous sections) can lead to the generation of integrated embryo models that contain both embryonic and extraembryonic cells. Remarkably, by starting solely with either totipotent-like mouse 2C-LCs or EPS cells, researchers have successfully generated mouse blastoids^87,234,235^. However, mouse EPS cell-derived blastoids generated controversy^95^. Gao lab found that the expression level of the trophoblast cell marker CDX2 in the TE-like structures of EPS-blastoids was much lower than that in the TE cells in normal blastocysts. In addition, the TE-like structures generally express specific genes of the PrE lineage, including GATA6, SOX17, and PDGFRα^236^. It is important to note that, not only in mice but also in the case of human cells, the resulting EPS-derived blastoids have the right morphology, but the cell types still differ from those of the actual blastocyst^237^. To further advance blastoid models, recent studies have reported the generation of human blastoids derived solely from reprogrammed fibroblasts^44^, human naive PSCs^43,238,239^ and from the recently reported totipotent-like human 8C-LCs^115^. Some attempts to form human blastoids resulted in the formation of structures morphologically similar to the blastocyst, consisting of an outer layer of cells called the trophectoderm enclosing a fluid-filled cavity containing a collection of cells called the ICM. However, subsequent analysis by an international consortium revealed that in several cases, the cellular states did not correspond to the blastocyst stage^240^. Human blastoids are considered a promising model of the human embryo, derived from cells cultured in vitro and aimed at containing all the founding cell lineages of the fetus and its supporting tissues theoretically^241^. As protocols are optimized, these blastoids will more closely mimic human blastocysts, with a reduction in the presence of off-target cells.

Currently, in the realm of human blastoid research, further optimizations have been made to the protocol for generating high-quality blastoids, addressing the issue of low initial efficiency^242,243^. This optimization enables the large-scale production of blastoids with epiblast-like cells, hypoblast-like cells, and trophoblast-like cells that more closely resemble the cell clusters and gene expression of human blastocyst. In addition, the research suggests that the endometrial stroma may promote the survival, proliferation, and cell fusion of trophoblast cells during co-cultivation with blastoids and endometrial stromal cells^242^.

The question of whether blastoids can recapitulate key features of human post-implantation development has been a subject of inquiry. Blastoids cultured on a 3D extracellular matrix can capture early post-implantation developmental characteristics^243^. These include the formation of the epiblast lumenogenesis, rapid expansion and diversification of trophoblast cell lineages, and increasing invasiveness by day 14. Prolonged culture of blastoids leads to localized activation of the primitive streak marker TBXT and the emergence of PGCs by day 21. The balance of epiblast and trophoblast cell fates in post-implantation blastoids can be modulated through the regulation of WNT signaling^243^. The blastoids have been developed to the gastrulation stage, further demonstrating their potential for continued development.

To circumvent ethical concerns associated with human embryo research, scientists have constructed monkey embryo-like structures resembling normal blastocysts using naive ESCs^116^. Through extended in vitro induction and cultivation, these blastoids develop structures similar to those found in normal embryos, including the yolk sac, chorionic cavity, amniotic sac, primitive streak, and connecting stalk. They exhibit morphology and cell lineages along the entire head–tail axis, which resemble those of typical embryos. Through single-cell transcriptomics or immunostaining, researchers have identified primitive germ cells, visceral endoderm/yolk sac endoderm, the three germ layers, and hematoendothelial precursors, among others. The transplantation of these blastoids into surrogate mothers results in implantation, but they do not develop further in vivo, indicating that their developmental potentiality is limited^116^.

Similarly, the team led by Jun Wu has utilized human extended pluripotent stem cells to self-organize into embryo-like structures within the WNT and Nodal signaling pathways, which are referred to as “peri-gastruloids“^4^ (Fig. 2). These structures comprise epiblast and hypoblast components. Although “peri-gastruloids” cannot survive without trophoblast cells, they recapitulate key stages of human embryonic development, including the formation of the amniotic sac and yolk sac cavity, the emergence of the two germ layers, primitive streak formation, the appearance of primordial germ cells, gastrulation, early neurulation, and organogenesis^4^.

## Discussion

The series of culture systems for mouse embryonic stem cells is notably extensive, and the depth of histological and developmental biological research on normal mouse embryos serves as a solid reference base for mouse embryo development models. In addition, the exploration of mouse in vitro culture systems has been comprehensive^244,245^, allowing for the construction of mouse embryo models using advanced in vitro culture systems that can develop to relatively later stages. In contrast, the study of early human embryonic development faces significant challenges due to difficulties in obtaining samples, the lack of clarity regarding the origin of extraembryonic tissues, challenges in the stable culture of extraembryonic cells, and the increasing ambiguity of embryonic structure and cell types as development progresses. These factors contribute to difficulties in developing human embryo models, which lack precise natural references. Simultaneously, although in vitro culture systems for primates have been continuously advancing in recent years^246–249^, they have their limitations, with normal embryonic development encountering obstacles that require continual adjustments and optimizations. In this context, these stem cells can be likened to “blocks” while the comprehensive understanding of the 3D spatial information and cell types of actual embryos serves as the “blueprint.” Effective utilization of these blocks, guided by the blueprint, is crucial for constructing embryo models that more closely resemble the in vivo conditions.

With the ongoing advancements in PSC research, it has become feasible to stably culture PSCs of various states in vitro, alongside the continual establishment of extraembryonic stem cell lines for a range of research objectives. Emerging stem cell-derived embryo models encapsulate certain characteristics of in vivo embryos, presenting profound significance, valuable application potential, and expansive developmental scope. These developments underscore three critical aspects: (1) the elucidation of developmental biological events through embryo models, (2) their utility in regenerative medicine, particularly in obtaining precursor cells for early organ development, (3) their potential in understanding diseases related to implantation failures, early miscarriages, and maternal–fetal interactions.

Although the establishment of embryo models plays a crucial role in providing an irreplaceable new perspective for studying early embryonic development, current embryo models still suffer from significant limitations. For instance, in vitro-derived embryo-like structures only partially recapitulate the characteristics of in vivo embryos, exhibiting substantial differences in lineage composition compared to in vivo counterparts. Some models lack complete extraembryonic tissues, possess intermediate unknown developmental lineages, and exhibit restricted posterior developmental potential with delayed development, among other issues. Furthermore, the construction of an embryo model cannot be achieved without an initial stable lineage. In terms of the induction and regulation of extraembryonic and embryonic cells, it is essential to conduct in-depth research on the signaling regulatory network mechanisms to stably control various cell lineages.

The future development of embryo models necessitates a focused approach on three principal aspects: refining the stem cell system, optimizing in vitro culture conditions, and addressing ethical considerations in-depth.

1. The current stem cell culture systems do not perfectly mirror the continuous process of cellular pluripotency observed in vivo, leading to discrepancies between in vitro stem cell states and their in vivo counterparts. Hence, it is crucial to promote the development of new stem cell lines and techniques that can more accurately capture and replicate the dynamic changes and state transitions of cells within the body. This entails the development of intermediate-stage stem cells and modulation of pivotal signaling pathways to derive both embryonic and extraembryonic stem cells, thereby encompassing the entirety of in vivo developmental stages. In addition, it is vital to consider the impact of epigenetics on the states of early embryonic stem cells to fully understand and mimic the complex biological processes involved^250^.
2. The optimization of in vitro culture systems is key to enhancing the quality of embryo models. Current culture conditions may not fully replicate the in vivo environment, resulting in cultured cells and tissues that deviate from their natural states within an organism. Future research efforts should be dedicated to developing more advanced culture media and techniques, such as 3D culture systems and bioreactors, as well as precisely controlling biochemical and physical conditions to more closely mimic the natural cellular growth environment, thereby improving the biocompatibility and functionality of cells and tissues. Alternatively, utilizing microfluidic technology to obtain embryo models with more complex structures.
3. As embryo models increasingly resemble real embryos, ethical issues become an unavoidable and critical concern. It is imperative to establish and refine ethical guidelines and legal frameworks to ensure that research does not overstep ethical boundaries. This includes setting clear limitations and regulations on the purposes, scopes, and methods of research using stem cells and embryo models, ensuring transparency and public right to information, and protecting the rights of patients and donors. Ongoing dialog and collaboration between ethicists, scientists, policymakers, and the public are also indispensable for finding a balance between scientific advancement and ethical principles.
